# Influence of Mineral Treatment, Plant Growth Regulators and Artificial Light on the Growth of Jewel Sweet Potato (*Ipomoea batatas* Lam. cv. Jewel) In Vitro

**DOI:** 10.3390/life13010052

**Published:** 2022-12-24

**Authors:** Rima N. Kirakosyan, Elena A. Kalashnikova, Halid G. Abubakarov, Nikolay N. Sleptsov, Yuliya A. Dudina, Sulukhan K. Temirbekova, Quyet V. Khuat, Vladimir I. Trukhachev, Anton V. Sumin

**Affiliations:** 1 Department of Biotechnology, Russian State Agrarian University—Moscow Timiryazev Agricultural Academy, Timiryazevskaya Street, 49, Moscow 127434, Russia; 2 Department of Plant Physiology, Russian State Agrarian University—Moscow Timiryazev Agricultural Academy, Timiryazevskaya Street, 49, Moscow 127434, Russia; 3 All-Russian Research Institute of Phytopathology, Bolshye Vyazyomy, Odintsovo District, Moscow 143050, Russia; 4 Department of Biology and Agricultural Engineering, Hanoi Pedagogical University 2, Nguyen Van Linh, Phuc Yen 15000, Vietnam; 5 Head Eployment, Russian State Agrarian University—Moscow Timiryazev Agricultural Academy, Timiryazevskaya Street, 49, Moscow 127434, Russia

**Keywords:** jewel sweet potato, shoot tip, axillary bud, different MS salts concentration, plant growth regulators, artificial light, micropropagation

## Abstract

Sweet potato (*Ipomoea batatas* (L.) Lam), a member of the bindweed family (*Convolvulaceae* Juss.), is well known for its food, medicinal, and industrial values. It is estimated that more than 7000 sweet potato cultivars have been bred to date. Jewel sweet potato (*I. batatas* Lam cv. Jewel) is one of the most popular cultivars of sweet potato grown today because of its high nutritional value, delicious taste, and is suitable for all processing methods. However, little is known about the micropropagation of jewel sweet potato. The purpose of this paper was to study the effect of three important factors, including culture medium, plant growth regulators (PGRs), and artificial light sources, on the induction, proliferation, and growth of in vitro *I. batatas* ‘Jewel’ shoots obtained from the axillary bud and shoot tip explants. The different Murashige and Skoog (MS) salt levels (33%, 50%, 100%, and 150%) were used to study the influence of mineral treatment. To assess the influence of PGRs, we used 0.5 mg/L indole-3-acetic acid (IAA) combined with various cytokinins, including 0.5–2.0 mg/L 6-benzylaminopurine (BAP), 0.5–2.0 mg/L kinetin (Kn), and 0.1–1.0 mg/L thidiazuron (TDZ). On the other hand, the in vitro shoots were cultivated in a light room with different lighting conditions. Three lighting treatments (differences in the ratio between the red (R) and blue (B) spectra) were used. Research results have shown that the medium containing 50% MS salt concentration supplemented with 0.5 mg/L BAP or 0.5 mg/L Kn combined with 0.5 mg/L IAA was the most suitable for induction, proliferation, and growth of in vitro jewel sweet potato shoots. On the other hand, stem pieces bearing the axillary buds’ explants were determined to be suitable for the shoot induction. Using artificial light with different blue/red ratios also had a significant effect on the growth of explants and stimulates shoot or root formation.

## 1. Introduction

Sweet potato (*Ipomoea batatas* (L.) Lam), a member of the bindweed family (*Convolvulaceae* Juss.), is well known for its food, medicinal, and industrial value [1,2,3]. More than 5000 years ago, this species was first domesticated in the Americas. It is estimated that more than 7000 sweet potato cultivars have been bred to date. Sweet potatoes are considered one of the most important root crops after potatoes and cassava [4], especially in developing countries in Latin America, Southeast Asia, and Africa [5]. In these countries, sweet potato is a main food crop for the people because of its richness with healthy proteins, vitamins, antioxidants, and minerals [6,7]. Studies have shown that this species possesses a number of pharmaceutical properties, such as antibacterial, antioxidant, anticancer, anti-inflammatory, and antiulcer activities [8,9,10]. In traditional medicine in many countries, sweet potato is used to treat diabetes, anemia, hypertension, stomach cancer, cardiovascular disease, allergies, constipation, eye disease, arthritis, dengue fever, and nausea [6,10,11,12,13,14,15]. On the other hand, sweet potato can be used in cooking, as well as in different industrial foods production [16,17,18].

Traditionally, sweet potato cultivars have been propagated by vegetative propagation using stem cuttings or tubers [7,19]. However, this method has certain limitations, such as slow propagation speed, being time-consuming, and being season-dependent. On the other hand, stem cuttings or tubers used for this method often accumulate pathogens (such as nematodes, insect pests, and pathogens that cause black rot, scurf, and stem rot) and can be spread from one generation to the next, causing great losses in yield and the production of poor-quality tubers [3,7,19]. Therefore, if large-scale, uniform, and disease-free plant material is required for production, this method is not always suitable [3,7]. On the other hand, the seeds of sweet potato are only used to breed and develop new cultivars of sweet potatoes because of their highly heterozygous nature [20]. In order to overcome the limitations, biotechnological methods using plant tissue culture techniques have been used for the commercial scale-up of many cultivars of sweet potato. In previous reports, several types of explants, e.g., nodal segment, axillary shoot, and shoot tip from mature or in vitro plants, have been used as explants for in vitro shoot proliferation of sweet potato cultivars [21,22,23,24,25,26,27,28,29,30,31,32,33,34,35,36,37,38,39]. In there, nodal segments have been recognized as the best or most commonly used explants for the micropropagation of various sweet potato cultivars, e.g., ‘Carmen Rubin’ and ‘White Triumph’ [32], ‘Gaozi No.1’ [27], ‘Naruto Kintoki’ [40], ‘KSP 36’ and ‘KEMB 36’ [41], and ‘Abees’ [4]. However, little is known about the micropropagation of jewel sweet potato, which is dubbed the ‘sweet potato queen’ due to its high nutritional value and delicious taste with all processing methods. This sweet potato cultivar was bred in 1970 at North Carolina State University. To date, jewel has become one of the most commonly grown sweet potato cultivars, its tubers are characterized by tan skin and orange flesh and that is considered a rich source of provitamin A carotenoid [42,43].

In fact, the efficacy of micropropagation techniques depends on a variety of factors, of which three important factors are culture medium, plant growth regulators (PGRs), and artificial light sources (type and intensity). In most reports, Murashige and Skoog (MS) [44] base medium was reported to be the most suitable medium for shoot initiation, shoot proliferation, and rooting in sweet potato cultivars [21,22,23,24,25,26,27,28,29,30,31,32,33,34,35,36,37,38,39]. However, in some sweet potato cultivars, 1/2MS medium has been found to be more suitable for shoot proliferation and rooting, e.g., ‘purple flesh sweet potato’ [37] and ‘red-peeled sweet potato’ [4]. Most of the authors have shown that the medium without any PGRs is not suitable for in vitro shoot regeneration. In sweet potato, nutrient media supplemented with cytokinins in combination with auxins were reported to be the best for in vitro shoot proliferation [21,22,23,26,37]. Besides the above two factors, the type and intensity of artificial light also affect the micropropagation of sweet potatoes. Yang et al. [45] found that the different ratios of photosynthetic photon flux (PPF) of red LED (R) and PPF of blue LED (B) had different effects on the growth of sweet potato plantlets in vitro.

The aim of the present study was to determine the effects of different PGRs, different MS salt concentrations, and different artificial light conditions on the induction, proliferation, and growth of in vitro *Ipomoea batatas* ‘Jewel’ shoots obtained from the axillary bud and shoot tip explants. 

## 2. Materials and Methods

### 2.1. Plant Material Preparation

The work was carried out at the Department of Biotechnology of the Russian State Agrarian University—Moscow Agricultural Academy named after K. A. Timiryazev (Moscow, Russia). All work on sterilization, introduction into culture in vitro, and further work on the study of callogenesis and morphogenesis were carried out in aseptic conditions of laminar hood flow (Biokom). The object of the study was explants of sweet potato (*Ipomoea batatas* Lam.), cultivar Jewel (USA), provided by the Federal Research Center for Potato named after A. G. Lorch.

Before being placed in vitro, the jewel sweet potato tubers were placed in water to activate the dormant meristem. On the 7th day, the shoots began to form, and on the 21st day, they reached 10–12 cm. The shoot tips of 1.0 cm in length and stem pieces bearing the axillary buds of 1.5 cm in length from plantlets sprouting from tubers were used as explants for experiments. The explants’ surface was sterilized with a 0.1% solution of mercuric chloride (HgCl_2_). They were soaked in the solution for 5 min and then rinsed with sterile distilled water [4].

### 2.2. Experiment 1: Evaluate the Influence of Different MS Salt Concentrations on Induction and Growth of In Vitro Jewel Sweet Potato Shoots 

The shoot tips of 1.0 cm in length and stem pieces bearing the axillary buds of 1.5 cm in length from plantlets sprouting from tubers were used as explants for this experiment. Nutrient media containing different MS salts (MS: DUCHEFA, Haarlem, The Netherlands) were created. The addition of 4.4 g/L MS was determined as 100%. The ratios of MS salts used in nutrient media: 33% (1.45 g/L-MS1), 50% (2.2 g/L-MS2), 100% (4.4 g/L-MS), 150% (6.6 g/L-MS3) [46]. Sucrose was present at a concentration of 2% and agar of 0.8% in all variants of nutrient media. PGRs were not added to the nutrient medium in all treatments. The pH of the nutrient medium in all treatments was 5.8. The plants were cultivated in a well-lit growth chamber at 21–23 °C under a 16 h photoperiod provided by 3–3.5 klx white fluorescent lamps (OSRAM AG, Munich, Germany).

In vitro shoot growth indices (including shoot length and root length) in the treatments were measured after one and four weeks of culture.

### 2.3. Experiment 2: Evaluate the Influence of Different PGRs on Proliferation and Growth of In Vitro Jewel Sweet Potato Shoots

Shoots 1.5–2.0 cm in length from the first experiment were used as explants for this experiment. The MS2 semisolid media supplemented with a combination of 0.5 mg/L indole-3-acetic acid (IAA) with various cytokinins, including 0.5–2.0 mg/L 6-benzylaminopurine (BAP) (Sigma, Schnelldorf, Germany), 0.5–2.0 mg/L kinetin (Kn) (Merck, Darmstadt, Germany), and 0.1–1.0 mg/L thidiazuron (TDZ) (Russia), were used to culture the in vitro shoots. There were 2% sucrose and 0.8% agar in all media. In vitro shoots were subcultured to a fresh medium every 6 weeks. Visual observations were made after 45 days. The following indicators were taken into account: explants’ survival rate, number of adventitious shoots per explant, shoot length, number of leaves per shoot, number of roots, and root length. The in vitro shoots were cultivated in a well-lit growth chamber at 21–23 °C under a 16 h photoperiod provided by 3–3.5 klx white fluorescent lamps (OSRAM AG, Munich, Germany). 

### 2.4. Experiment 3: Evaluate the Influence of Different Artificial Light Conditions on Induction and Growth of In Vitro Jewel Sweet Potato Shoots 

The shoots 1.5–2.0 cm in length from the first experiment were cultivated on two different types of media:

(1) PGR-free MS medium containing sucrose 2% and agar 0.8%;
(2) Medium containing only distilled water and agar 0.8%, without mineral salts and PGRs.

The in vitro shoots were cultivated in a light room with different lighting conditions. Three lighting treatments (differences in the ratio between the red (R) and blue (B) spectra) were used: 

Treatment 1 (control): illuminators based on white LEDs with a color temperature of 3500 K and 6000 K and a monochromatic red LED with a peak of 660 nm (OSRAM AG brand, made in Germany).

Treatment 2a: multichannel illuminator based on white LEDs with a color temperature of 3500 K and 6000 K and monochromatic red (R) and blue (B) LEDs with peaks of 660 nm and 460 nm, respectively. The channel power of monochromatic LEDs was set in the ratio R: 70%/B: 30%.

Treatment 3a: multichannel illuminator based on white LEDs with a color temperature of 3500 K and 6000 K and monochromatic red and blue LEDs with peaks of 660 nm and 460 nm, respectively. The power of monochromatic LED channels was set in the ratio R: 30%/B: 70% [47].

In vitro shoot growth indices (including shoot length, root number, and root length) in the treatments were measured after 45 days of culture. 

### 2.5. Statistical Analysis of Experimental Data

The experiments were arranged completely randomly and repeated three times. Mean values of all data were calculated using Microsoft Excel 2013 (Microsoft Corporation, Redmond, WA, USA). Analysis of variance (ANOVA) was performed in AGROS software (version 2.11, Russia) and means were compared using Fisher’s least significant difference (LSD) test at a significance level of *p* ≤ 0.05.

## 3. Results

### 3.1. Influence of Different MS Salt Concentrations on Induction and Growth of In Vitro Jewel Sweet Potato Shoots

The induction and growth of in vitro shoots from explants depend on the amount of salts dissolved in the medium. In this experiment, the standard MS salt composition was changed to 33% (MS1), 50% (MS2), 100% (MS), and 150% (MS3) of the normal concentration (100% MS).

**Figure 1:**
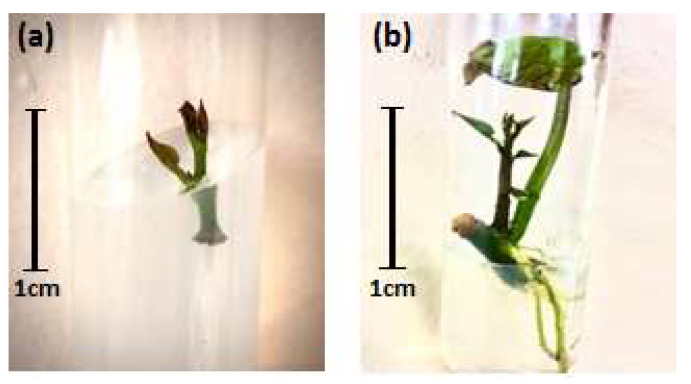
Response of the explants after 7 days of culture: (**a**) shoot tip; (**b**) stem pieces bearing the axillary bud.

After 7 days of culture, the shoot tip explants showed no response to the culture medium (Figure 1a), while the stem pieces bearing the axillary buds showed to form the axillary shoots and roots (Figure 1b). Observations in the following weeks did not record the response of shoot tip explants.

**Figure 2:**
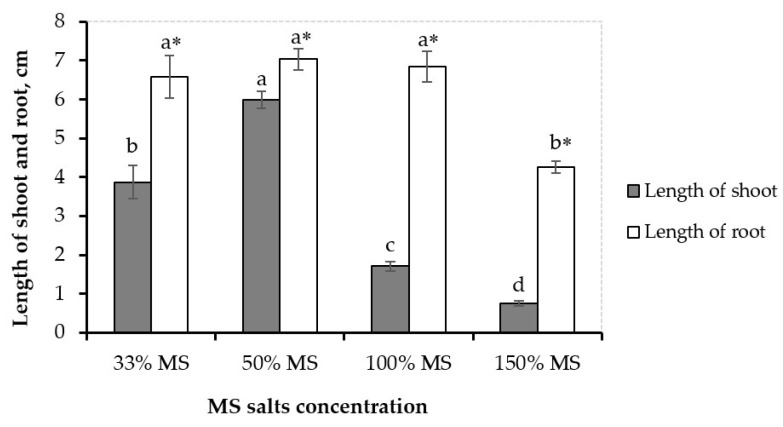
Influence of different MS salt concentrations on in vitro shoots’ growth of jewel sweet potato after four weeks of culture. (*) indicate a significant interaction between the evaluated parameter at 0.05 probability levels. Means with different letter(s) within the bars differ significantly at a 0.05 probability level using LSD.

On the other hand, the results also showed that the medium containing different concentrations of MS salts had a significant effect on the growth of axillary shoots and roots of sweet potato after four weeks of culture. The reduction in salt content compared with basal MS medium showed good results for the sprouting of axillary buds, as well as axillary shoots’ subsequent growth. The best growth indices (including axillary shoot length and root length) were obtained from explants on the MS2 medium (Figure 2). High concentrations of mineral salts in the culture medium (MS3) showed a negative effect on axillary shoots’ and roots’ growth. Based on these obtained results, MS2 medium (containing 50% salt content of the salt concentration in basal MS medium) was used for the following experiments.

### 3.2. Influence of Different PGRs on Proliferation and Growth of In Vitro Jewel Sweet Potato Shoots

**Figure 3:**
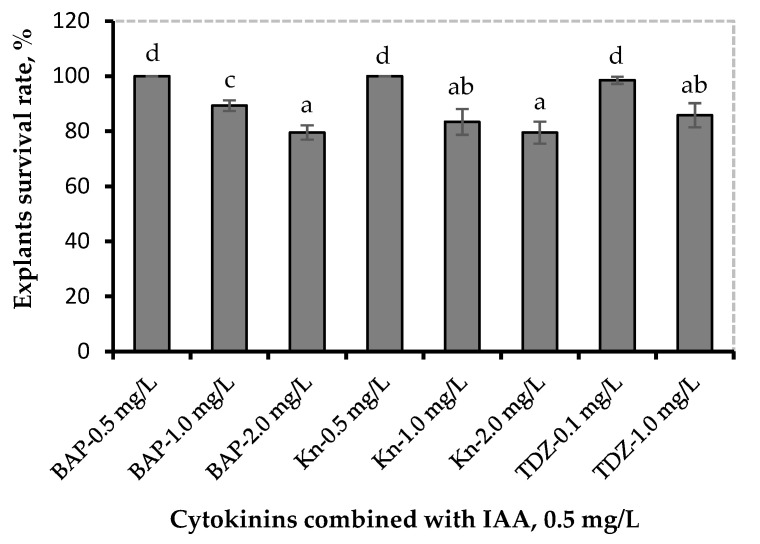
Combined effect of auxin and cytokinin on explants survival rate (%) after 45 days of culture. Values of germination percentage were arcsine √X transformed prior to statistical analysis. Means with a different letter(s) within the bars differ significantly at a 0.05 probability level using LSD.

The MS2 medium (containing 50% salt content of the salt concentration in basal MS medium) supplemented with a combination of 0.5 mg/L IAA with various cytokinins such as 0.5–2.0 mg/L BAP, 0.5–2.0 mg/L Kn, and 0.1–1.0 mg/L TDZ was used for the rapid proliferation of in vitro jewel sweet potato shoots (Table 1, Figure 3).

The obtained results demonstrate that increased cytokinin concentration in the nutrient medium reduced the explants’ ability to form axillary and adventitious shoots, whose growth was also reduced due to higher hormonal concentrations. It was found that the best results were obtained in the medium containing 0.5 mg/L BAP and 0.5 mg/L Kn combined with 0.5 mg/L IAA, and the worst in the medium containing TDZ combined with 0.5 mg/L IAA. The media with BAP combined with 0.5 mg/L IAA exhibited average results (Table 1).

Differences were also observed for such parameters as shoot length and the number of leaves per shoot (Table 1). The experiments demonstrated that the MS2 medium supplemented with 0.5 mg/L Kn combined with 0.5 mg/L IAA provided the highest mean shoot length value (6.1 cm) and the number of leaves per shoot (9.2). The second effective medium was one containing 0.5 mg/L BAP combined with 0.5 mg/L IAA; its shoot length was 5.2 cm, and the number of leaves was 8.0. In the other medium, the in vitro shoots grew slowly and formed very short internodes, which was especially evident for the medium containing 1.0 mg/L TDZ combined with 0.5 mg/L IAA.

In addition, the combined addition of cytokinin and auxin (IAA) showed the in vitro rooting effect. The results showed that the MS media supplemented with 0.5 mg/L BAP and 0.5 Kn combined with 0.5 mg/L IAA gave the highest number of roots and mean root length (0.5 mg/L BAP: 2.02 roots/shoot, 8.95 cm; 0.5 mg/L Kn: 1.98 roots/shoot, 8.52 cm).

### 3.3. Influence of Different Artificial Light Conditions on Induction and Growth of In Vitro Jewel Sweet Potato Shoots 

In this experiment, we studied the effect of the spectral composition of the light (red and blue spectrum) on the in vitro shoots’ growth of jewel sweet potato. The main research results are shown in Table 2. Studies have shown that the addition of red and blue spectra in different proportions to normal illumination did not lead to an increase in the growth of cultivated explants. As a rule, the specific growth rate of the main shoot from axillary buds was about 2–2.5 times less than in the control variant.

According to the obtained results, studied treatments had an ambiguous influence on the growth indices of jewel sweet potato shoots. Within the nutrient medium containing MS salts, the studied indicators were less or equal to the control variant. The exception was observed at the R = 30%: B = 70% treatment. In this treatment, the mean number of roots per shoot was 5.25, which is about 1.75 times higher than in the two other treatments.

As regards cultivation of in vitro shoots on medium free of mineral salts with only water, sucrose, and agar, clearer dependences were observed. Under these conditions, the mean shoot length was maximal at the R = 70%: B = 30% treatment. At the R = 30%: B = 70% treatment, the mean number of roots was 5.67, which was nearly 2 times more than in other treatments within the used medium. 

When growing in vitro shoots of jewel sweet potato on a nutrient medium without mineral compounds, an inverse relationship was observed between the mean number of roots and the proportion of red and blue spectra. There was an increase in root formation as a result of the increase in the blue spectrum proportion. Shoot growth was observed with the predominance of the red spectrum. It can be seen that, by changing the composition of light, it is possible to regulate the morphogenetic potential of jewel sweet potato.

## 4. Discussion

Like most European countries, the sweet potato cultivation area in the Russian Federation is limited, concentrated mainly in southern provinces. In recent years, one of the popular trends in the food industry is the manufacturing of functional and dietary food products. Only in Russia, about 1400 tons of such products are consumed annually, and most of these are imported. The practical requirement is to expand the area of material plants for this industry and sweet potato is one of them. In the present study, we selected the ‘Jewel’ sweet potato cultivar, which has high nutritional value and is popularly grown in many countries around the world, to study the factors affecting their in vitro propagation ability, thereby creating a premise to expand large-scale production in the direction of gradually replacing imported raw materials for the food industry. 

Investigations in the field of plant cell engineering start from a well-grown sterile culture. Many publications have demonstrated that the proper selection of a sterilizing agent, its concentration, and its effect on an explant are vital parts of a study that in many ways determine the success of an experiment [48]. To obtain a sterile sweet potato culture, many authors applied 0.1% HgCl_2_ solution to soak the explants for 14–15 min [49,50,51]. However, such a long exposure may cause necrotic lesions in the young and actively growing plant tissues, leading to their premature death. Our experiment, performed in plants of different taxonomic groups, showed that the best sterile explants were obtained from the plant tissues socked in 0.1% HgCl_2_ solution for 5 min. Similarly, Dewir et al. [4] also obtained good surface sterilization of red-peeled sweet potato explants using 0.1% HgCl_2_ solution for 5 min.

The success of clonal micropropagation depends on the balanced composition of the nutrient medium, both in terms of mineral and PGR composition. Several reports on other plant species have demonstrated that different MS salt concentrations affect the growth development or regeneration values of plants, such as *Mentha spicata* L. [52], *Bacopa monnieri* L. [53], *Lophophora williamsii* Coult. [54], and *Staurogyne repens* (Nees) Kuntze [46]. In sweet potato, the MS base medium was reported to be the most suitable medium for shoot initiation, shoot proliferation, and rooting in most reports [21,22,23,24,25,26,27,28,29,30,31,32,33,34,35,36,37,38,39]. However, in some sweet potato cultivars, 1/2MS medium has been found to be more suitable for shoot proliferation and rooting, e.g., ‘purple flesh sweet potato’ [37] and ‘red-peeled sweet potato’ [4]. The results of our study on the sweet potato jewel cultivar also showed that the medium containing 50% MS salt was the most suitable for shoot initiation, shoot proliferation, and rooting. On the other hand, most of the reports also showed that the addition of BAP or Kn combined with IAA resulted in good shoot regeneration and rooting effects [21,22,23,26,37]. Similar to these reports, our results suggest that a nutrient medium supplemented with 0.5 mg/L BAP or 0.5 mg/L Kn combined with 0.5 mg/L IAA was best for shoot initiation, shoot proliferation, and rooting in sweet potato jewel cultivar.

It is known that the spectral composition of light is an important physical factor influencing morphogenetic processes. It was shown that different light spectra affect the proliferation and differentiation of plant cells in different ways. For example, violet and blue spectra increase the process of photosynthesis, which leads to the rapid formation of more powerful plants [55]. Plant photomorphogenesis depends on the intensity of the red and blue spectrum of light, as well as their ratio. It has been experimentally shown that the spectrum of red light is quite wide. Its different parts are responsible for the regulation of various physiological processes. This may affect the production process as a whole [55]. In addition, the synthesis of auxins depends on red light. Auxins are responsible for root differentiation in an intact plant. The blue spectrum is responsible for the differentiation of buds and the formation of the aboveground biomass. The green spectrum leads to an increase in the effectiveness of the action of various spectra on the morphophysiological processes of the studied objects [56,57]. The results of our study have shown that an increase in the proportion of the blue spectrum stimulates an increase in root formation. The predominance of the red spectrum stimulates the activation of shoot growth. Due to the fact that very few similar studies have been reported previously, present and future studies in this direction are of interest.

## 5. Conclusions

This study is one of the first to report the effects of culture conditions on the micropropagation of *Ipomoea batatas* ‘Jewel’ cultivar. Effects of three important factors (including MS salt concentration, PGRs, and artificial light) on the initiation, proliferation, and growth of in vitro jewel sweet potato shoots obtained from the axillary bud and shoot tip explants were studied. Research results have shown that the medium containing 50% MS salt concentration supplemented with 0.5 mg/L BAP or 0.5 mg/L Kn combined with 0.5 mg/L IAA was the most suitable for induction, proliferation, and growth of in vitro jewel sweet potato shoots. This result will pave the way for further studies on the jewel sweet potato cultivar towards determining the biochemical composition, especially inulin content, biological activity, and adaptability to field conditions in the Russian Federation of plantlets. Thereby creating a premise to expand large-scale production in the direction of replacing imported raw materials for the food industry to meet practical needs.

## Figures and Tables

**Table 1:** Combined effect of auxin and cytokinin on proliferation and growth of in vitro jewel sweet potato shoots after 45 days of culture.

Plant Growth Regulator, mg/L		Number of Shoots per Explant	Shoot Length	Number of Leaves per Shoot	Number of Roots per Shoot	Root Length
Cytokinin	Auxin		(cm)			(cm)
BAP, 0.5 mg/L	IAA, 0.5 mg/L	3.2 ± 0.2 ^1^ d	5.2 ± 0.2 b	8.0 ± 0.3 b	2.02 ± 0.12 a	8.95 ± 0.75 a
BAP, 1.0 mg/L		1.8 ± 0.5 b	4.0 ± 0.2 c	6.6 ± 0.3 c	1.51 ± 0.51 ab	8.06 ± 1.02 abc
BAP, 2.0 mg/L		1.6 ± 0.3 b	3.0 ± 0.1 d	3.0 ± 0.1 d	0.86 ± 0.26 bc	7.68 ± 0.65 bc
Kn, 0.5 mg/L		3.9 ± 0.1 d	6.1 ± 0.3 a	9.2 ± 0.4 a	1.98 ± 0.07 a	8.52 ± 0.38 ab
Kn, 1.0 mg/L		2.6 ± 0.1 c	4.3 ± 0.2 c	5.1 ± 0.2 c	1.35 ± 0.36 abc	8.01 ± 0.52 abc
Kn, 2.0 mg/L		2.0 ± 0.4 bc	3.3 ± 0.2 d	2.9 ± 0.1 d	1.01 ± 0.65 bc	7.51 ± 0.36 bc
TDZ, 0.1 mg/L		1.5 ± 0.2 b	2.6 ± 0.1 de	3.0 ± 0.1 de	1.25 ± 0.14 bc	7.23 ± 0.45 c
TDZ, 1.0 mg/L		0.8 ± 0.1 a	0.7 ± 0.1 f	2.5 ± 0.1 f	0.65 ± 0.43 c	5.86 ± 0.14 d

^1^ Mean ± standard error (SE), means followed by the same letter are not significantly different at *p* ≤ 0.05 according to the Fisher’s least significant difference (LSD) test.

**Table 2:** Influence of the ratio of red and blue spectra on growth of in vitro jewel sweet potato shoots after 45 days of culture.

Medium Type	Light Treatment	Number of Shoots per Explant	Shoot Length, (cm)	Number of Roots	Root Length, (cm)
PGRs-free MS medium	Control	1	4.06 ± 1.32 ^1^ e	3.75 ± 0.15 c	10.25 ± 0.69 c
	R 70%: B 30%	1	2.95 ± 0.54 d	3.25 ± 0.16 b	9.75 ± 0.60 c
	R 30%: B 70%	1	1.80 ± 0.12 d	5.25 ± 0.25 d	9.87 ± 0.63 c
Distilled water and agar 0.8%	Control	1	0.72 ± 0.10 a	2.25 ± 0.11 a	7.62 ± 0.38 b
	R 70%: B 30%	1	1.33 ± 0.10 c	2.33 ± 0.15 a	7.33 ± 0.33 ab
	R 30%: B 70%	1	0.95 ± 0.10 b	5.67 ± 0.38 d	6.83 ± 0.30 a

^1^ Mean ± standard error (SE), means followed by the same letter are not significantly different at *p* ≤ 0.05 according to Fisher’s least significant difference (LSD) test.

## Data Availability

Not applicable.
